# An Optimised Spider-Inspired Soft Actuator for Extraterrestrial Exploration

**DOI:** 10.3390/biomimetics10070455

**Published:** 2025-07-11

**Authors:** Jonah Mack, Maks Gepner, Francesco Giorgio-Serchi, Adam A. Stokes

**Affiliations:** School of Engineering, Institute for Integrated Micro and Nano Systems, The University of Edinburgh, The King’s Buildings, Edinburgh EH9 3LJ, UK; j.mack-1@sms.ed.ac.uk (J.M.); m.gepner@ed.ac.uk (M.G.); f.giorgio-serchi@ed.ac.uk (F.G.-S.)

**Keywords:** soft robotics, bio-inspired design, space exploration, jumping locomotion, spider-inspired actuators, hydraulic actuation, 3D printing, extraterrestrial robotics, untethered robots, TPU actuators

## Abstract

Extraterrestrial exploration presents unique challenges for robotic systems, as traditional rigid rovers face limitations in stowage volume, traction on unpredictable terrain, and susceptibility to damage. Soft robotics offers promising solutions through bio-inspired designs that can mimic natural locomotion mechanisms. Here, we present an optimised, spider-inspired soft jumping robot for extraterrestrial exploration that addresses key challenges in soft robotics: actuation efficiency, controllability, and deployment. Drawing inspiration from spider physiology—particularly their hydraulic extension mechanism—we develop a lightweight limb capable of multi-modal behaviour with significantly reduced energy requirements. Our 3D-printed soft actuator leverages pressure-driven collapse for efficient retraction and pressure-enhanced rapid extension, achieving a power-to-weight ratio of 249 W/kg. The integration of a non-backdriveable clutch mechanism enables the system to hold positions with zero energy expenditure—a critical feature for space applications. Experimental characterisation and a subsequent optimisation methodology across various materials, dimensions, and pressures reveal that the robot can achieve jumping heights of up to 1.86 times its body length. The collapsible nature of the soft limb enables efficient stowage during spacecraft transit, while the integrated pumping system facilitates self-deployment upon arrival. This work demonstrates how biologically inspired design principles can be effectively applied to develop versatile robotic systems optimised for the unique constraints of extraterrestrial exploration.

## 1. Introduction

### 1.1. Soft Robotics for Extraterrestrial Exploration

There is significant interest in extraterrestrial exploration, particularly towards Mars and the moons of Jupiter, from both private and public sectors. Current approaches have utilized rigid rovers for planetary exploration, but these suffer from critical limitations, including constrained stowage volume, limited traction on uneven or granular terrain, and vulnerability to mechanical damage. Ishigami et al. demonstrated the sinking terramechanics of rover wheels interacting with Martian soil [1], underscoring the need for alternative mobility strategies. Soft-legged locomotion offers a promising solution to the challenges of wheeled motion on complex planetary surfaces such as Mars [2]. Feng et al. demonstrated a compact cubesat-style robot employing tendon-driven retraction and soft compliant hinges for deployment, highlighting the energy efficiency of jumping locomotion in low-gravity environments [3]. A compelling example of the energy benefits of such locomotion is the transition of Apollo astronauts from walking to hopping gaits on the lunar surface.

In addition, pneumatic actuation, which is prevalent in soft robotics, is inherently compatible with the low atmospheric pressures of extraterrestrial environments. The pressure differential between internal actuation chambers and the ambient environment enhances actuator performance and reduces energy requirements, as described by Ng and Lum [4]. The material properties of thermoplastic polyurethane (TPU) further strengthen the case for soft robotic systems in these contexts. TPU maintains its elasticity at sub-zero temperatures, making it particularly advantageous for Martian applications where surface temperatures can regularly fall below −60 °C. As shown by recent studies [5,6], TPU exhibits robust mechanical performance and durability even in low-temperature and low-pressure environments. These properties support the use of TPU-based limbs and joints in extraterrestrial soft robots, ensuring reliable deformation and recovery cycles despite harsh environmental conditions. Moreover, TPU’s flexibility and impact resilience reduce the likelihood of material fatigue or failure under repeated high-strain activities such as jumping or terrain negotiation, making it an ideal candidate for long-duration missions.

### 1.2. The Difficulty of Untethering Soft Robots and the Compromises of Soft Actuation

One of the foundational benefits of soft robots is their ability to mimic natural systems [7,8,9]. The actuation methods prevalent in soft robotics closely resemble those found in nature, in contrast to rigid robots that rely primarily on electromagnetic actuation [10,11]. Despite these similarities, there is a significant difference in the efficiency with which input energy is utilized. According to a review by Shui et al., mobile soft robots generally have an efficiency of only 0.1% [12]. This low efficiency can be attributed in part to ineffective mechanical conversion or the reliance on multi-stage power-conversion trains. Energy losses are a critical factor in the systems-level design of robots. Notably, the gap between the energetic efficiency of terrestrial animals and robots is smaller for rigid robots than for soft robots [13,14]. Chun et al. highlight the importance of systems-level design, rather than a component-level approach, using a bond-graph theory-based abstraction [15]. Their abstraction emphasizes the need for a holistic approach to soft robot design, considering the entire system rather than individual components, especially for untethered soft robots.

Making untethered soft robots is difficult due to two main factors: (1) dependence on heavy external pneumatic or electric systems [16], and (2) inefficiencies in the design and manufacture of soft actuators. These inefficiencies mean that system design choices for a soft robot are made later in the process. Ideally, the system requirements would inform the rational choice and design of the actuator. This actuator-first approach means only a few untethered jumping soft robots exist. Tolley et al. built an untethered explosion-driven jumping soft robot capable of directional jumps of 0.6 m [17], but the use of explosives limits the repeatability and operational safety. Other jumping robots, such as the JelloCube created by Li and Rus, demonstrate dynamic bounding movements by using a spring winding and release mechanism to enable rapid actuation [18]. The use of compression springs allows for large jumps but limits the controllability of actuation. These robots demonstrate two approaches to enable dynamic movement and highlight the difficulty in achieving the levels of adaptability exhibited by animals. Such methods of actuation involve a compromise between actuation rates and controllability.

Furthermore, the difficulty of analytically modelling the complex material behaviours and non-linear mechanical responses often renders purely analytical approaches insufficient. As a result, the geometry and material choices for many soft actuators are not optimised for use in an end system. This experimental foundation enables us to develop a practical optimisation methodology informed by system-level requirements rather than being constrained by theoretical limitations.

Considering the constraints of extraterrestrial exploration, such as the inclusion of sensor packages, an untethered robot would need to be able to locomote with a payload. Sayed et al. demonstrate a 10 g sensor payload capable of environmental sensing for extreme environments [8]. To fully actualise the potential benefits of soft robots for space exploration applications, actuation needs to be compact, controllable, and energy-efficient.

### 1.3. Spider Bio-Mechanics Offer a Framework for Efficient Soft Systems

**Figure 1:**
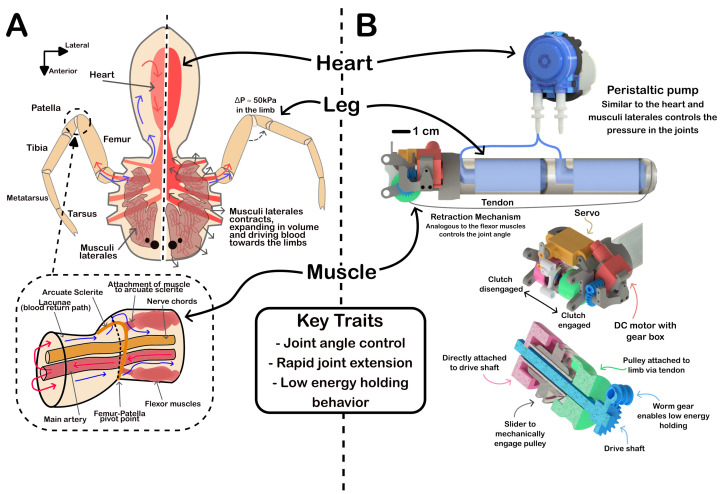
(**A**) Cartoon demonstration of the key hydraulic features of a spider: red arrows show oxygenated hemolymph and blue arrows show deoxygenated hemolymph travelling back through empty spaces inside the spider. (**B**) The rendering on the right of the figure demonstrates the function of the proposed actuator, highlighting the inspiration drawn from the function of the spider to develop the actuator. The STEP file needed to view the retraction mechanism in 3D is available at https://github.com/Soft-Systems-Group/Spider-Inspired-Soft-Jumping-Actuator accessed on 30 May 2025.

One candidate for energetic actuation in soft robots is the hydraulic extension mechanism found in arachnids. Arachnids generally lack extensor muscles in their legs [19,20]. Manton and Harding proposed that this hydraulic extension evolved to allow limbs to be lightweight, enabling energy-efficient locomotion [21]. These limbs require only a single set of muscles per joint, but—through the use of auxiliary systems—remain fully functional. Hydraulic extension is driven by muscles in the prosoma (body), which pump hemolymph (blood) into the cavities of the leg (lacunae, Figure 1A). We used spider morphology as a blueprint for designing our soft robot, highlighting the benefits of hybrid systems.

Göttler et al. have published seminal work investigating *Phidippus regius* (the jumping spider) and the subsequent application of its extension mechanisms in robotics [22,23,24]. Notably, a fully 3D-printed soft thermoplastic polyurethane (TPU) folding actuator that was able to jump with manual retraction and a pressure system tether was designed [23]. Spider-inspired designs have been used to reduce the internal friction of hydraulic systems; for example, the rotary actuator presented by Hepp and Badri-Spröwitz. Landkammer et al. used fluidic extension and fluidic muscle contraction to create a rigid spider-inspired limb. Designs that have taken inspiration from but do not closely mimic arachnida have also been explored [25,26,27,28,29], but the work conducted by Göttler et al. is the only soft spider-inspired actuator to demonstrate tethered jumping behaviour [23]. Our work extends this by exploiting the benefits of vacuum-driven collapse in combination with a lightweight retraction system to develop a first-of-its-kind untethered soft robotic jumping.

If a spider wishes to move more dynamically, then it can increase the effective stiffness or spring constant of its limb by tensing muscles in the prosoma, which, in turn, close off the blood return path (see Figure 1A). The build-up of pressure inside the limb drives the leg to a straightened position when the flexor muscles are relaxed. Due to the variability of the material stiffness in the limb sections, most of the energy from the fluid is utilised at the point of articulation. The pressurised blood pushes the more flexible membranes of the joint outward, minimising wasted energy in attempts to extend away from the joint. In non-dynamic cases, the muscles in the prosoma are relaxed and the blood can flow freely back to the body. We draw inspiration from the hybridisation of the systems by selectively engaging specific features of the system to improve the overall efficiency.

### 1.4. A Spider-Inspired Thin-Membrane Soft Actuator Which Exhibits Efficient and Dynamic Actuation with Minimal Control Inputs

As discussed in Section 1.1 and Section 1.2, the design of untethered soft robots presents core challenges, but also offers key benefits for extraterrestrial exploration. To mimic the dynamic and efficient movement of natural systems, we combine bio-inspiration with the unique advantages of soft robotics to demonstrate a lightweight, energy-efficient actuation system for space applications.

In this work, we demonstrate the benefits of combining auxiliary systems in an untethered soft robot. As shown in Figure 1, we seek to mimic the key traits of the spider’s movement mechanics. Furthermore, with a focus on space applications, we aim to develop a lightweight, energy-efficient actuation system that can be used in space exploration. Thus, the key design specifications are as follows: (1) exhibit control of a joint angle; (2) exhibit dynamic or rapid extension for jumping; (3) exhibit low energy-holding behaviour; and (4) be lightweight and volume-efficient. Achieving these specifications would make the actuator a compelling candidate for space exploration applications compared to purely electro-mechanical or purely soft pneumatic systems.

We refer to the proposed actuator as “JUMPER”—Joint Unbuckling Mechanism with Pressure-enhanced Extension and Retraction. The actuator is shown in Figure 1B and consists of two key components: a TPU thin-membrane 3D-printed cylinder and a clutched wind and release mechanism.

**Figure 2:**
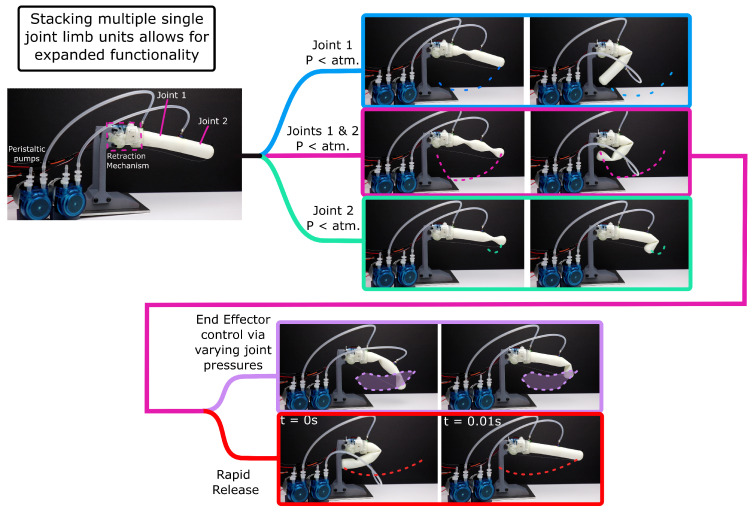
Demonstration of the multimodal behaviour of a single JUMPER actuator and how stacked joints can exhibit a range of complex angle configurations. A video demonstrating the multi-modal behaviour of the multi-jointed JUMPER system can be found here: https://vimeo.com/1089032212/70b518941e accessed on 30 May 2025.

We designed JUMPER’s soft component to be simple and relatively easy to print. Single-joint variants can be easily printed on many desktop 3D printers and can be stacked to create multi-jointed cylinders. The use of Fused Deposition Modelling (FDM) printing allows for rapid prototyping and consistent wall thickness in the cylinder. Printed multi-jointed cylinders, such as the one shown in Figure 2, would pose a significant challenge for traditional 3D-printing methods; for this reason, we utilised the Flex Printer, an open-source 3D printer designed for the development of soft robots [30]. We capitalised on pressure-driven collapse to reduce the effective stiffness of the cylinder, allowing for more efficient retraction.

**Figure 3:**
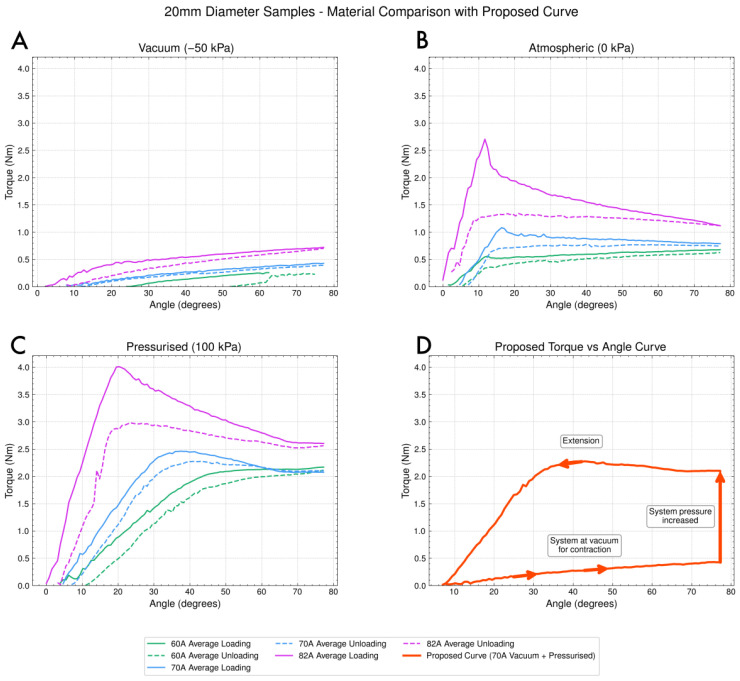
(**A**–**C**) Loading and unloading behaviour of the 20 mm diameter cylinder at different pressures for different materials, showing the significantly reduced loading force for vacuum conditions. (**D**) The proposed actuation cycle, where the cylinder is at vacuum for retraction then pressurised for extension.

The reduction in force used to retract the joint in turn reduces the energy consumption of the retraction mechanism for locomotion or manipulation. During retraction, the geared DC motor draws 0.175 watts of power. The motor itself draws 0.15 watts of power when disconnected from the system. The motor is unable to retract the joint at atmospheric pressure (stalling at around 0.4 watts). As shown in Figure 3A, the force required to retract the joint at vacuum is roughly half that of the force required to retract the joint at atmospheric pressure. Due to the non-backdriveable nature of the worm drive, the cylinder can be at vacuum for retraction and then pressurised for extension (shown by Figure 3D). This non-backdriveability allows us to exploit the low force of loading under vacuum, and then, via pressurisation, jump to the unloading behaviour of the 100 kPa curve. By using the auxiliary pump system, similar to the spiders musculli laterales, a single pump can control the pressure in multiple cylinders or joints. Such a design benefits from upscaling, where a single pump and fluid-routing system can enhance the performance of multiple actuators. In instances where multiple joints are required, the pressure system can be used to control the behaviour of the limb, as demonstrated in Figure 2. The cylinder itself also serves multiple functions, it is a single-component joint not requiring any bearings or axles, and simultaneously serves as the structure of the limb.

The retraction system was designed to fulfil the criteria we described previously. A small DC motor drives a worm gear, the output of which is attached to a servo-controlled clutch mechanism. The worm gear allows the actuator to hold a position with zero energy expenditure. The ability to hold a position indefinitely enables unique traits, particularly in the context of space exploration. The servo-controlled clutch mechanism draws virtually no energy while idle; when engaged, the clutch connects the worm gear to the retraction pulley (see Figure 1), allowing for specific joint angle control for retraction and extension. To achieve dynamic jumping, the pressure in the joint can be increased while retracted, and the clutch can be disengaged to allow the joint to extend freely and rapidly.

## 2. Materials and Methods

As mentioned in Section 1.1, untethered jumping soft robots offer a promising approach for extraterrestrial exploration. To create such a robot, the diameter, material, and pressure of a single spider joint must be determined. To identify the optimal joint configuration under specific system constraints, we employ a combination of experimental sweeps and parameterised optimisation techniques. Our goal is to maximise actuator impulse while minimising system mass. Four main metrics are considered: (1) the force produced over the actuator’s range of motion (unloading work, ULW); (2) the rate at which the actuator can rotate (extension rate, ERT); (3) the volume change required to transition from evacuated to a given pressure (total volume change, TVC); and (4) the force required by the retraction motor to wind the evacuated cylinder to 85 degrees (vacuum retraction force, VRF).

Göttler et al. presented a spider-inspired limb that has a diameter of 20 mm [23]. Tested samples by Hugo and Lee had a length to width ratio of 2.5–5 (15 mm to 30 mm diameters), as a result, the diameters of 16 mm, 20 mm, 24 mm, and 28 mm were selected [31]. Each sample had a length of 50 mm, with 4 mm of solid-filled end pieces for mounting and for the tube inlet. The PLA-printed rigid mounts were epoxied to the test sample to ensure a complete bond. The 6 mm tube was inserted into the sample as a press fit; although this provides a useable seal, epoxy was used to ensure there was no leakage. The samples were tested from vacuum to 1.5 Bar. Many of the samples were more than capable of being pressurised to over 2.5 Bar (250 kPa,  36 psi), but the risk of rupture would increase significantly. The system works with both air and water, but for the experiments we used water such that the compressibility of air did not impact the results.

**Figure 4:**
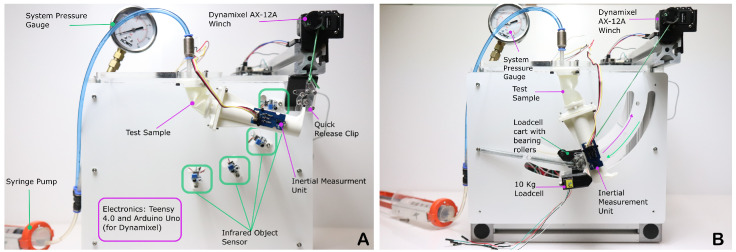
The experimental setup to determine the speed of the sample via time between trigger signals, and an IMU, with a 600 MHz processor (**A**), and the setup to determine peak force and work with a 10 kg loadcell on a circular arc track (**B**).

As shown by Figure 4, we constructed two experimental setups to determine the four main parameters. The setup used to determine the retraction and extension force utilised a winch driven by a Dynamixel AX-12A (Dynamixel, Seoul, Korea). The winch pulls on a bearing roller cart, which holds a load cell (RS part: 283-6585, Sparkfun, Niwot, USA). The load cell then applies force to the tip of the cylinder sample being tested. We conducted these tests for a range of pressures, from vacuum to 1.5 bar, at 0.25 bar increments. In the firmware of the microcontroller, we applied a low pass filter to the load cell output. We determined the angle of the cylinder using the BMI-088 inertial measurement unit (RS part: 245-7082, Bosch, Reutlingen, Germany). The orientation of the sample was obtained through resolving the gravity vector and fusing it with the integrated gyro angle via a complementary filter. The springs that are visible in the test setup were to help overcome and mitigate friction in the roller during the extension/unwinding phase.

### 2.1. Determining the Peak Retraction Force and Unloading Work

To determine the peak retraction force, we increased the system volume via the syringe pump until the system reached −0.5 Bar. We then began an automated test procedure. The automated procedure begins winding the string at a rate of 50 RPM. This winding pulls the roller cart up the track and the system measures the load via the load cell.

We found the loading and unloading force vs. angle by winding the sample up to 85 degrees and then unwinding at a constant rate. The unloading work over the full sweep of 85 degrees is useful to determine the energy that can be converted into jumping. Due to the aforementioned complexities in the pre- and post-buckling phases, for use in a jumping robot, having a large range of motion and understanding the force behaviour over this range of motion is important. For use cases where a smaller range of motion is desired, the actuator configuration could be selected such that a peak force is provided at or around a specific range of motion.

### 2.2. Determining the Angular Rate

The experimental setup we used to determine the extension rate utilises four infrared object-detecting sensors (LM393-based sensor, Dallas, USA), spaced equally at 22.5-degree increments. These sensors were connected to a Teensy 4.0 (RS part: 283-6910, Sparkfun, Niwot, USA), which was selected for its clock cycle speed. We determined, using a high-speed camera, that the rate of extension would grow close to the maximum measurable rate of the BMI-088 IMU, and that the highspeed camera would not have the necessary refresh rate to accurately capture the total time. The sample would be pressurised to the desired test pressure, the AX-12A winch would then retract the sample to 85 degrees, and the sample would then be released via the quick-release clip. The IMU would record the angular rate and acceleration of the sample, and the IR sensors would record the time from the point at which first sensor could no longer detect the sample. The resulting speeds between the two intervals were averaged to provide the rate.

## 3. Results

**Figure 5:**
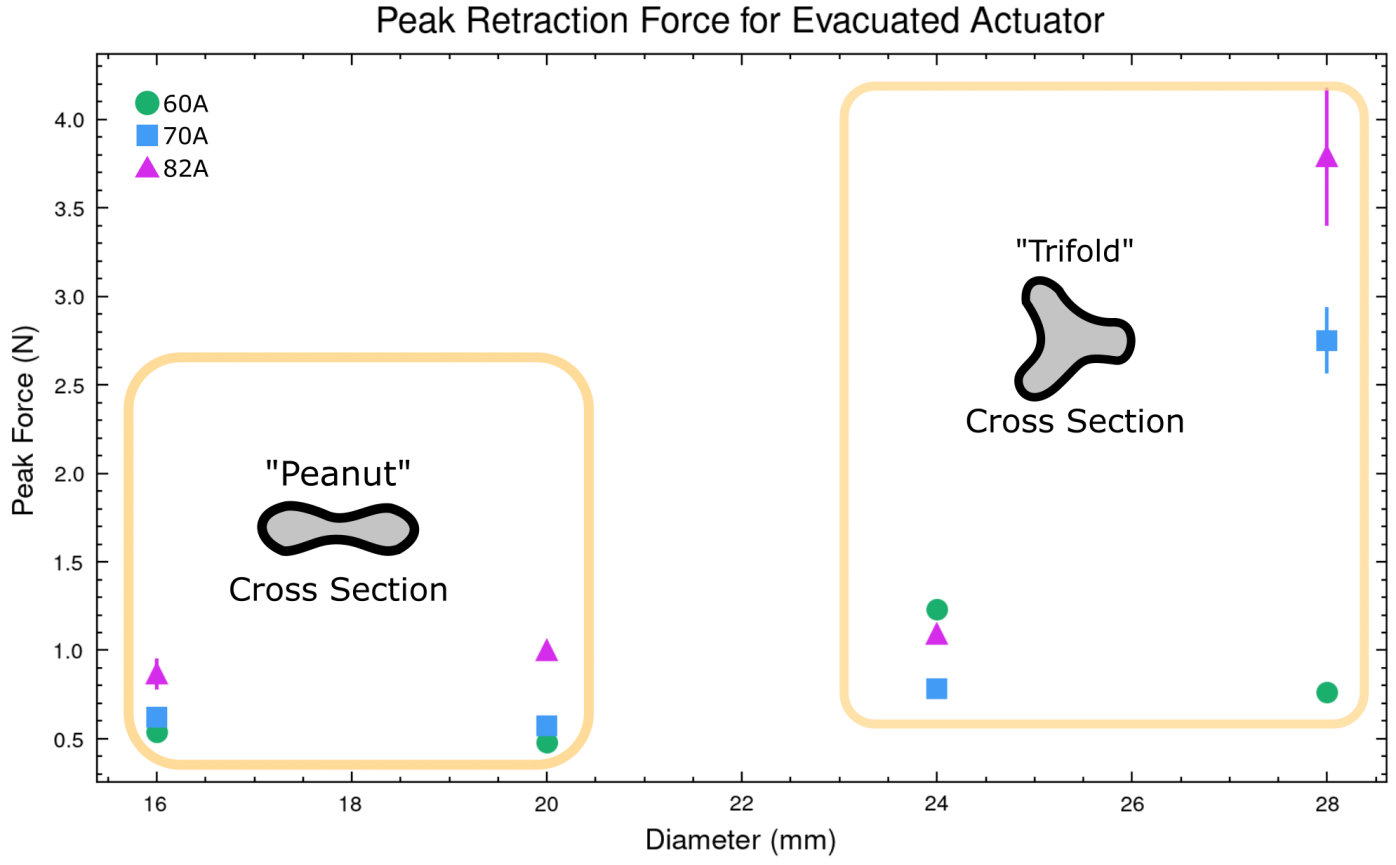
Peak measured retraction force for the evacuated cylinder with observed differences in cross-sections.

The vacuum retraction force was expected to be primarily a function of material stiffness. The crumpled “peanut” shape shown in Figure 5 reduces the cross-section available for loading. While there is extensive work on the failure modes of hollow cylinders under external pressure—such as that by Timoshenko and Gere [32]—there is significantly less research on the collapse of hyperelastic materials. Due to the complexity of various collapse modes, recent studies have employed finite element analysis (FEA). Zhu et al. were able to simulate the observed behaviour we refer to as “trifolding,” where, as demonstrated in Figure 5, the cross-section at larger diameters exhibits unexpected collapse behaviour [33]. The data indicate that trifolding increases the loading force.

This complexity is why an experimentally driven selection approach was utilised for the VRF. The “trifolding” behaviour introduces unwanted uncertainty around the point of rotation of the actuator. In some instances, the “trifolding” would snap into a “peanut” cross-section; this was the case with the 28 mm 60 A sample. The extension behaviour did not seem to be dramatically impacted by the “trifolding”, but trifolding is important to consider in cases where a specific rotation axis is desirable, such as ours. We also note an interesting phenomenon: the samples that trifold often exhibited a rotation around the longitudinal axis of the cylinder, decreasing their total length.

The speed and unloading experiments that were conducted allowed for an investigation of the output power of the various samples. The unloading curve over the 85-degree sweep and the total time to extend can be combined to provide a single power value for each actuator configuration.

**Figure 6:**
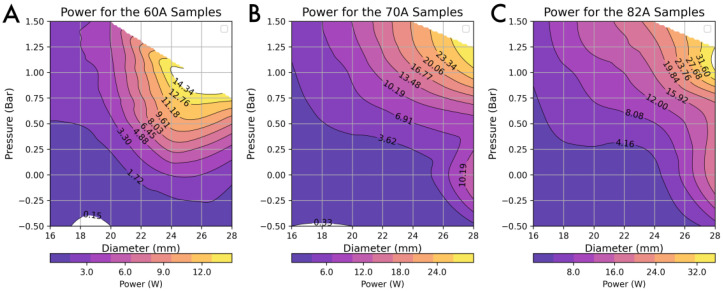
Contour plot of the power during the extension phase of 85 degrees of the tested samples across the three materials. Each sample of varying diameter and material was tested at each pressure three times (note: the colour scale is different for the three contours).

In Figure 6, we can observe that the power increases with an increase in the diameter and pressure of the samples. We attribute this to larger diameters and stiffer materials producing more force over the range of angles. We find that pressurisation of the cylinder acts as a means of introducing energy to the system and, as demonstrated in the theory highlighted by Micklem et al. [34], increases the resistance to buckling. We note that the data presented in Figure 6 aligns with our first principle that increasing the pressure and second moment of area an alongside increase in material stiffness increases the output power. However, the contour plots show that the relationship is not entirely linear, and we identify this as highlighting the complex interplay of material damping and pressure behaviours.

### Optimisation to Select an Ideal Configuration Based on System Heuristics

Section 3 indicates that the behaviour of the single-joint system deviates from the theory, making it difficult to purely analytically optimise the configuration. As discussed in Section 1.2, our approach aims to avoid the shortcomings of analytical or simulation-based optimisation through rapid iterations of a single soft joint. The experimental setup was designed such that the key parameters relevant to the task of jumping can be isolated and parameterised. We can then reduced each actuator into four key parameters and assigned constraints to the optimisation such that the system informs the choice.

For a single joint of JUMPER, we can reduce each combination of material, diameter, and pressure to four parameters: vacuum retraction force (VRF), total volume change (TVC), unloading work (ULW), and extension rate (ERT). These are the key parameters to optimise. For each configuration, we normalised the experimental results per parameter (VRF, TVC, ULW, ERT) from −10 to 10. A “weight” was assigned to each parameter, representing its importance in the overall system. Each weight had a value between 0 and 10, where 0 means the parameter is not important and 10 means the parameter is very important. The range of weights or normalization is arbitrary, as the relative ranking is what determines the selection.

Such an abstraction allows for a refinement of the desired configuration and allows for a heuristic approach to resolving a multi-parameter problem. The pseudo-code for the optimisation procedure is shown in Algorithm 1 (the optimisation code is also available: https://github.com/Soft-Systems-Group/Spider-Inspired-Soft-Jumping-Actuator accessed on 30 May 2025.)
**Algorithm 1** Calculate maximum score from multiple normalised configurations:  1:**procedure** MaxScoreFromConfigs  2:      maxScore←0  3:      $\mathrm{weights}\leftarrow [w_{1},w_{2},w_{3},w_{4}]$                 ▹ Define weight vector  4:      **for** each set∈Configurations **do**  5:            VRF,TVC,ULW,ERT←set  6:            params←[VRF,TVC,ULW,ERT]  7:            normalisedParams←normalise(params,−10,10)  8:            score←0  9:            **for** i←1 to 4 **do**10:                  score←score+normalisedParams[i]×weights[i]11:            **end for**12:            **if** score>maxScore **then**              ▹ Check for new max score13:                  maxScore←score14:            **end if**15:      **end for**16:      **return** maxScore17:**end procedure**

**Figure 7:**
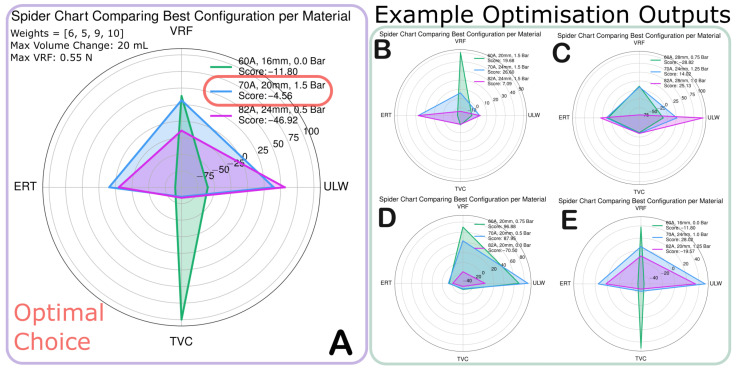
The visualisation output from the optimisation algorithm, demonstrating the “best in class” configuration for the three materials. (**A**) shows the output for the proposed system; (**B**–**E**) show example outputs with various weights applied.

Based on this optimisation, the parameters for the force of the retraction motor (Figure 1) and the total driveable volume of the selected peristaltic pump were applied to the set of configurations in the optimiser. As shown in Figure 7A, the optimal choice is the 20 mm 70 A durometer cylinder. Figure 7B shows some alternative examples of what the output may look like for various systems to demonstrate how various weights can impact the results. The key benefit of the presented visualisation is that the strengths and weaknesses of the configuration are clearly displayed and compared to other close-scoring configurations. The ability to enter different system constraints and to receive a summary of the best possible configurations demonstrates the potential for soft roboticists to make more informed system decisions.

Informed by the system constraints and the results of the optimisation, we inserted the selected cylinder into a simple, two-legged, untethered spider robot. We designed the robot to serve as a proof of concept for the optimisation and to demonstrate that we can successfully implement the retraction mechanism in a lightweight untethered robot for space exploration applications. We measured the fully assembled robot with its two 300 mAh 1S lipo batteries as having a mass of 197.36 grams.

**Figure 8:**
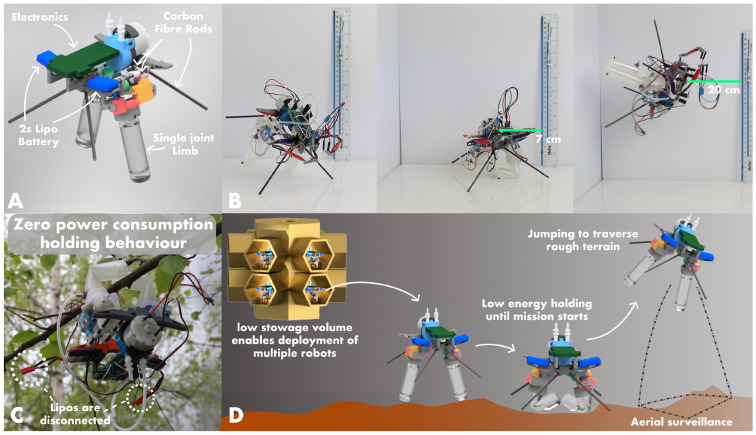
(**A**) The main components of the full robot utilizing two single-unit JUMPER actuators. (**B**) The robot performs a controlled extension and an untethered jump. A video can be found here: https://vimeo.com/1089032212/70b518941e (Supplementary Materials). (**C**) The robot can maintain retracted positions indefinitely and without power; here, we show the robot holding onto a tree branch without power, demonstrating the extended lifetime of operation. (**D**) A cartoon render illustrating a potential implementation of the simple robot for aerial surveillance and exploration over rough terrain on extraterrestrial surfaces.

As shown by Figure 8B, the simple two-legged robot is capable of jumping. Over eight jumping trials, the robot was able to jump an average of 1.86 its body length when comparing its stowed length (floor to centre of mass) to its average vertical displacement. The multi-modal controlled retraction and extension (shown by Figure 2), alongside the untethered jumping behaviour described above, makes JUMPER a first-of-its-kind soft robot. The jumping cycle consists of four distinct steps. The pump runs for 20 s, drawing 1.8 watts of power to evacuate the two cylinders. The motors then retract the limbs for 10 s, each drawing 0.175 watts. The peristaltic pump motor then pressurises the limbs for 1 min to reach 1 bar, consuming the same power as was used to evacuate the limbs. The two servo motors disengage the clutch system (drawing 0.5 watts each during this operation). As shown in Figure 6, the 70 A 20 mm cylinder at 1 bar produces 7.39 watts of mechanical power. For the two limbs, this would provide a total of 14.78 watts of total mechanical output power. From these values and the measured extension speed of 0.044 s for the 70 A sample, the energy-efficiency is calculated as 0.39%, providing a nearly 4× improvement compared to the average efficiency of many soft robots according to Shui et al. [12]. This improvement in efficiency highlights the benefit of a "V-model" in system design, drawing from nature and incorporating multiple modes of operation to optimise performance [15]. This approach allows for the more efficient use of energy and resources, leading to improved overall system performance.

## 4. Discussion

The JUMPER actuator represents a significant advancement in soft robotics, successfully combining bio-inspired design principles with practical engineering solutions for extraterrestrial exploration. The spider-inspired hydraulic extension mechanism provides a compelling framework for achieving both precise control and dynamic actuation within a single system. Our experimental results demonstrate that, by manipulating internal pressure, we can effectively switch between controlled positioning and rapid jumping modes—addressing the fundamental challenge of balancing controllability and dynamic performance in soft robotics. Table 1 highlights the contribution of the JUMPER actuator to the existing soft and rigid robotic literature.

The optimisation methodology presented offers a systematic approach to soft actuator design that moves beyond trial-and-error methods. By parameterising key performance metrics and implementing a weighted scoring system, designers can make informed decisions based on specific application requirements. This approach is particularly valuable for space applications, where weight, power consumption, and reliability constraints are paramount.

The observed “trifolding” behaviour, while initially presenting challenges for consistent rotation axis control, reveals interesting opportunities for future exploration. This phenomenon could potentially be harnessed for novel actuation modes or multi-axis movement capabilities, expanding the versatility of the actuator design.

The energy-efficiency improvement of nearly 4× compared to typical soft robots (0.39% vs 0.1%) demonstrates the potential of hybrid actuation systems. While still lower than rigid robotic systems, this represents a significant step towards bridging the efficiency gap between soft and traditional robotics. The ability to hold positions with zero energy consumption through the non-backdriveable worm gear mechanism provides additional advantages for space applications where power conservation is critical.

Future work should explore scaling effects, multi-actuator coordination, and integration with advanced sensing systems for autonomous operation in extraterrestrial environments. The modular design of JUMPER enables its straightforward integration into larger robotic systems, making it a promising candidate for future space exploration missions.

## 5. Conclusions

This paper introduced JUMPER (Joint Unbuckling Mechanism with Pressure-enhanced Extension and Retraction), a novel spider-inspired actuator that exhibits unique multi-modal behaviour—the first of its kind to combine controlled extension/retraction with a dynamic jumping capability. By drawing inspiration from spider physiology, we demonstrated how bio-inspiration can be effectively translated into practical soft robotic systems that address the core challenges of untethered soft robotics: power density, control complexity, and energy-efficiency. We show how the single-joint unit can be extended for complex locomotion behaviour, or used more simply in a small untethered robot.

Our approach utilises rapid prototyping through 3D printing to systematically explore and optimise the design space across multiple parameters—material stiffness, cylinder diameter, and operating pressure—enabling a practical, experiment-driven optimisation methodology. This approach circumvents the limitations of analytical or simulation-based methods when dealing with the complex non-linear behaviours of soft materials under varying pressure conditions. The resulting actuator demonstrates significant performance improvements when compared to existing soft jumping mechanisms, exhibiting controlled motion during normal operation while maintaining the ability to rapidly deploy stored energy for jumping.

Drawing inspiration from spider hydraulic mechanisms, JUMPER achieves a significant advancement in soft robotic actuation efficiency by switching between different operational modes. Our experimental results demonstrate that pressure modulation enables direct control over both actuator stiffness and dynamic response characteristics, seamlessly transitioning between precise positioning and high-energy jumping behaviours within a single device. This dual-mode functionality positions JUMPER as an ideal candidate for extraterrestrial exploration missions, where the combination of adaptability, reliability, and energy-efficiency is essential for mission success.

Furthermore, the parametric optimisation methodology we presented provides a framework for designing application-specific soft actuators that can be tailored to meet the constraints of various deployment scenarios. The visualisation of multiple performance metrics—simultaneously—enables designers to make informed trade-offs between competing objectives, a critical capability for the development of practical untethered soft robots.

JUMPER demonstrates that combining bio-inspiration with systematic experimental optimisation can yield soft robotics solutions that overcome many of the traditional limitations of the field. The lightweight, energy-efficient design, coupled with its multi-modal operation capabilities, positions this actuator as a promising technology for future extraterrestrial exploration missions, where adaptability and robustness are essential.

## Figures and Tables

**Table 1:** Comparison of jumping robots: BL = Body Length.

Robot	Jump (BL)	Mass (g)	Non-Jumping Locomotion	Actuation
This work	1.86	197.3	Y	Electric + Pneu.
Göttler et al. [23] ^a^	∼1.25	∼200	N	Electric + Pneu.
Tolley et al. [17]	∼7.5	510	N	Explosion
JelloCube [18]	∼7.6	90	N	Electric + Spring
MIT Cheetah [35]	∼1.5	∼27,000	Y	Electric
Mini Jumper [36]	27	7	N	Electric + Spring
Light-Powered [37]	5	25	Y	Light/Phase

^a^ Not untethered—uses external pneumatic supply.

## Data Availability

The data, optimisation algorithms, JUMPER CAD files, and figure generation code are openly available at https://github.com/Soft-Systems-Group/Spider-Inspired-Soft-Jumping-Actuator accessed on 30 May 2025.

## Supplementary Materials

The following supporting information can be downloaded at: https://vimeo.com/1089032212/70b518941e accessed on 30 May 2025.

## Abbreviations

The following abbreviations are used in this manuscript:

JUMPER	Joint Unbuckling Mechanism with Pressure-enhanced Extension and Retraction
TPU	Thermoplastic Polyurethane
FDM	Fused Deposition Modelling
VRF	Vacuum Retraction Force
TVC	Total Volume Change
ULW	Unloading Work
ERT	Extension Rate
FEA	Finite Element Analysis
IMU	Inertial Measurement Unit
